# ToF-SIMS 3D imaging unveils important insights on the cellular microenvironment during biomineralization of gold nanostructures

**DOI:** 10.1038/s41598-019-57136-w

**Published:** 2020-01-14

**Authors:** Ajay Vikram Singh, Harald Jungnickel, Lars Leibrock, Jutta Tentschert, Philipp Reichardt, Aaron Katz, Peter Laux, Andreas Luch

**Affiliations:** Department of Chemical and Product Safety, German Federal Institute for Risk Assessment (BfR), Max-Dohrn-Strasse 8-10, 10589 Berlin, Germany

**Keywords:** Cell biology, Materials science, Nanoscience and technology

## Abstract

The biomolecular imaging of cell-nanoparticle (NP) interactions using time-of-flight secondary ion mass spectrometry (ToF-SIMS) represents an evolving tool in nanotoxicology. In this study we present the three dimensional (3D) distribution of nanomaterials within biomolecular agglomerates using ToF-SIMS imaging. This novel approach was used to model the resistance of human alveolar A549 cells against gold (Au) ion toxicity through intra- and extracellular biomineralization. At low Au concentrations (≤1 mM HAuCl_4_) 3D-ToF-SIMS imaging reveals a homogenous intracellular distribution of Au-NPs in combination with polydisperse spherical NPs biomineralized in different layers on the cell surface. However, at higher precursor concentrations (≥2 mM) supplemented with biogenic spherical NPs as seeds, cells start to biosynthesize partially embedded long aspect ratio fiber-like Au nanostructures. Most interestingly, A549 cells seem to be able to sense the enhanced Au concentration. They change the chemical composition of the extracellular NP agglomerates from threonine-*O*-3-phosphate aureate to an arginine-Au(I)-imine. Furthermore they adopt the extracellular mineralization process from spheres to irregular structures to nanoribbons in a dose-dependent manner with increasing Au concentrations. The results achieved regarding size, shape and chemical specificity were cross checked by SEM-EDX and single particle (sp-)ICP-MS. Our findings demonstrate the potential of ToF-SIMS 3D imaging to better understand cell-NP interactions and their impact in nanotoxicology.

## Introduction

Au nano- or microparticles are promising candidates for both bioimaging and therapeutic applications^1^. Recently, cell based biomineralization has been explored as a novel technique to synthesize versatile 0, 1, and 2 dimensional gold nanostructures under ambient synthesis conditions. For example, cancer cells are known to synthesize NPs de novo^2^ from Au ions. However, little is known about how the surrounding chemical environment influences the extracellular biomineralization at the cell membrane interface and what effect this then has on uptake and biocompatibility.

Time of Flight - Secondary Ion Mass Spectrometry (ToF-SIMS) is a versatile and noninvasive technique that allows for both label free imaging and analysis of the surface composition. Conventional scanning electron microscope-energy dispersive X-ray (SEM-EDX) imaging or fluorescence labelling of biological cells not only perturbs the cellular state but also limits the analysis to a specific number of fluorescence labeled targets. On the contrary, ToF-SIMS provides a more versatile method for the visualization of multiple chemicals or intracellular metabolites within individual cells^3^. The submicron lateral resolution in combination with a high depth resolution for organic samples (down to 10 nm^3^) enables the three dimensional (3D) label-free reconstruction of single cells. This makes ToF-SIMS a top emerging and evolving tool for studies relating to the chemical distribution within single cells^4^.

Here we apply ToF-SIMS 3D imaging to map the intracellular and protein-mediated biomineralization of gold (Au) ions into spherical and anisotropic Au nanoparticles (Au-NPs) in A549 cancer cells under controlled conditions. Chemical imaging through ToF-SIMS highlights significant biochemical differences between the *de novo* synthesized NP structures^2^. To date, 3D imaging of nanoribbon-like long-aspect ratio anisotropic structures have never been realized in biological matrices so far^5^. Here we envision the biological synthesis of two dimensional (2D) nanoribbons in the presence of toxicologically relevant lung alveolar A549 cells.

We demonstrate the combination of SEM-EDX and ToF-SIMS imaging to assess the chemical composition of the de novo synthesized nanostructures (spheres, irregular shaped particles and nanoribbons). This approach will enable semi-quantitative, marker-free, simultaneous analysis of all cell membrane components while cells undergo biochemical changes at different Au ion precursor concentrations. The data generated by ToF-SIMS can also be used for 3D reconstruction of distribution patterns of nanofibers within single cells. The combination of ToF-SIMS, X-ray spectroscopy and scanning microscopy reveals the fine structure and the biomolecular distribution that cannot be seen by conventional techniques in such a non-invasive manner.

## Results

### ToF-SIMS 3D imaging of human alveolar epithelial cells growing in a mild chemical environment

**Figure 1:**
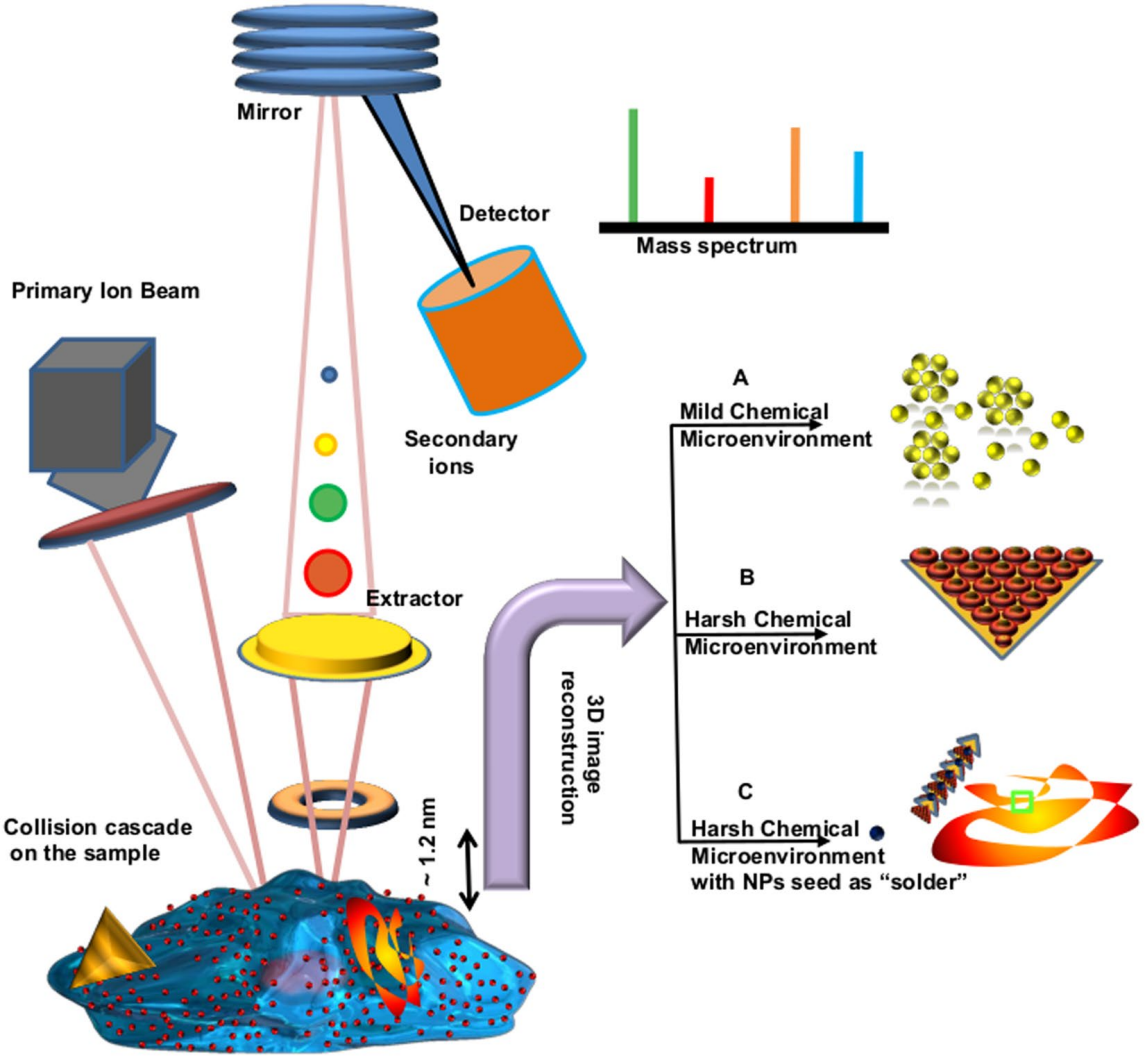
The schematic overview of the workflow of the 3D biomolecular imaging of de novo biomineralization of ionic gold into anisotropic (0, 1 & 2D) nanostructures. Left panel show the collection of secondary ions by the detector after the primary ion beam impacts freeze dried A549 cells with embedded anisotropic gold nanostructres. The right panel shows that ToF-SIMS images can be reconstructed into 3D space to give molecular distributions of gold and reducing agent in three different culture environments resulting in 3 different nanostructures (spheres, irregular particles and nanoribbons).

As shown in Fig. 1 right panel (A–C), we investigated three different biomineralization environments with lung alveolar A549 cells under serum free conditions: (A) HAuCl_4_ at low concentration (0.5 mM) (B) HAuCl_4_ at high concentration (2 mM), and (C) 2 mM AuCL4 with pre-synthesized 20 nm spherical Au-NPs as seeds. With the low concertation (A) of AuCl A549 cells reduced Au ions to spherical Au-NPs. With higher concentrations delta/rhombus shaped microplates were formed through nucleation-seed growth. Finally the addition of seeds (C) changed the synthesized particles to high aspect ratio nanowires. The size and zeta potential of the nanostructres formed in different chemical environments are listed in Table 1.

**Table 1:** DLS/zeta potential measurement of different nanostructres biomineralized by A549 cells.

	Spherical particles	Quasi-spherical particles	Rhomboid particles (largest face)	Nanoribbons (width/length)
Size (nm)	60 nm	75 nm	1–3 µm	50 nm/9 µm
Zeta potential (mV)	−13.5	−9.21	−14.09	−6.44

**Figure 2:**
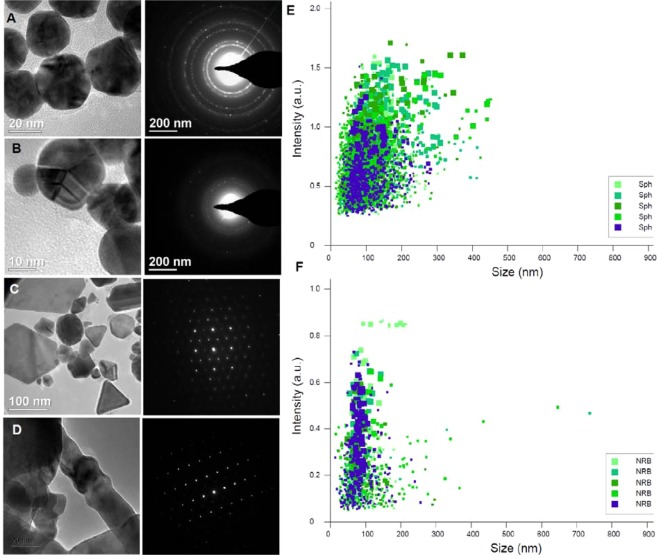
High resolution transmission electron microscopy (HR-TEM) and nanoparticle tracking analysis (NTA) of biomineralized AuNP materials. The Brownian motion and light scattering information from NTA gives the size of distribution and HRTEM and shape of nanostructures formed by A549 cells treated with (**A**) 0.5 mM; (**B**) 1.0 mM; (**C**) 2.0 mM; and (**D**) 2.0 mM HAuCl_4_ with spherical Au-NPs as seeds. (**E**,**F**) NTA plots showing size distribution after 5 rounds of tracking of spherical NPs (**E**) corresponding to scheme (**A**) and nanoribbons (**F**) corresponding to (**C**) of right panel in Fig. 1.

**Figure 3:**
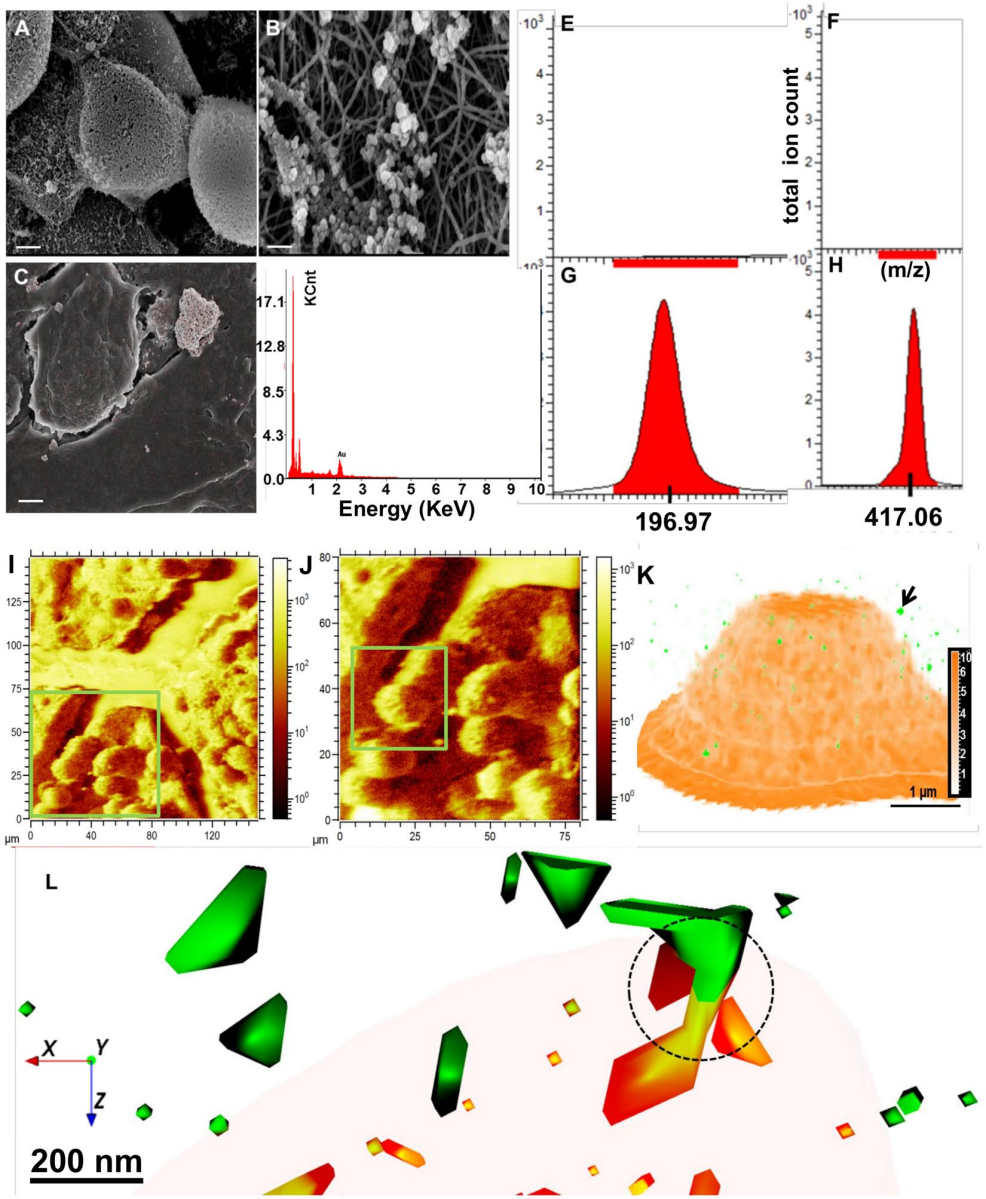
Scanning electron microscopy and ToF-SIMS molecular 3D imaging of condition (**A**) in schematic Fig. 1. Scanning electron microscopy (**A**,**B**) and X-ray spectroscopic (**C**,**D**) analysis of A549 cells treated with 0.5 mM HAuCl_4_ confirms NP formation (scale bar 5 µm in **A,C** and 200 nm in **B**). The ion signals from Au^+^ (**E**, untreated A549 cells; **G**, ionic gold) and threonine-*O*-3-phosphate aureate (**F**, untreated A549 cells; **H**, A549 cells treated with low dose of ionic gold). (**I**,**J**) Topography of extracellular spherical Au nanostructures on top of A549 cells (square ROI 130 µ × 130 µ in I and 80 µ × 80 µ in J). (**K**) Reconstructed 3D depth profile of mineralized Au structures in A549 cell cultures. The Au^+^ signal (red, translucent) indicates the spherical Au-NP, whilst the signal for threonine-*O*-3-phosphate aureate is shown in green. (**L**) Enlarged view from (**K**, arrow) showing the combined distribution of threonine-*O*-3-phosphate (green) and Au-NPs signals (orange) with occasional overlap (ring).

The formation of spherical Au-NPs indicates an inherent ability of A549 cells to detoxify their cellular environment. This is confirmed by TEM/SEM imaging, X-ray spectroscopy and NTA as shown in Figs. 2 and 3A–D. The Au ions are biomineralized into spherical NPs on the cell membrane and can be seen on the cell surface in Fig. 3A. This is even more clearly seen where Au is embedded into the actin cytoskeleton micro/nanofibres (Fig. 3B). this result is in line with our recent reports on breast cancer cell-mediated biomineralization^2^. The SEM-EDX shows accumulated Au signals emanating from Au clusters on the cell’s surface (Fig. 3C,D).

The SEM can only provide information about the surface layers. What occurs inside different NP layers, and what the chemical composition of the biomineralized cluster is composed of, is a difficult task to address when using only conventional techniques. This report highlights the novel capability of ToF-SIMS to obtain in-depth 3D chemical information within Au-NP structures embedded in a single A549 cell. The ToF-SIMS acquired data shows an Au+ signal (m/z 196.17) associated with Au+ containing bioorganic matrices. A simultaneous signal from de novo synthesized threonine-*O*-3-phosphate aureate complexed Au (m/z 417.06) was detected. None of the above ions were detected in untreated control cells (compare Fig. 3E,F vs G,H).

For the analysis of de novo biosynthesis of Au-NPs, first a dataset with a wide field of view (130 µm × 130 µm) was recorded where many cells could be observed (Fig. 3I,J). 3D reconstruction of a tighter 60 µm × 60 µm depth profile enables the 3D visualization of Au particles. Images were reconstructed from the Au^+^ signal (red, translucent) and the threonine-*O*-3-phosphate aureate (green) network as shown in Fig. 3K. Data shown for the m/z 196.97 (Au^+^ in Fig. 3G), shares a similar distribution with threonine-*O*-3-phosphate aureate (green, Fig. 3K). Further zoom-in reveals that the threonine-*O*-3-phosphate aureate signal is primarily localized on the outside surface of the Au particle. A small amount is localized within the de novo synthesized Au particle. One half of the threonine-*O*-3-phosphate agglomerate is situated within and the other half remain outside the Au particle (Fig. 3L). The m/z signals emerging threonine-*O*-3-phosphate aureate and Au^+^ are seen overlapping in the center of the nanoparticle agglomerate as observed in and around the edge of the signals shown with ROI (ring, 3.L).

**Figure 4:**
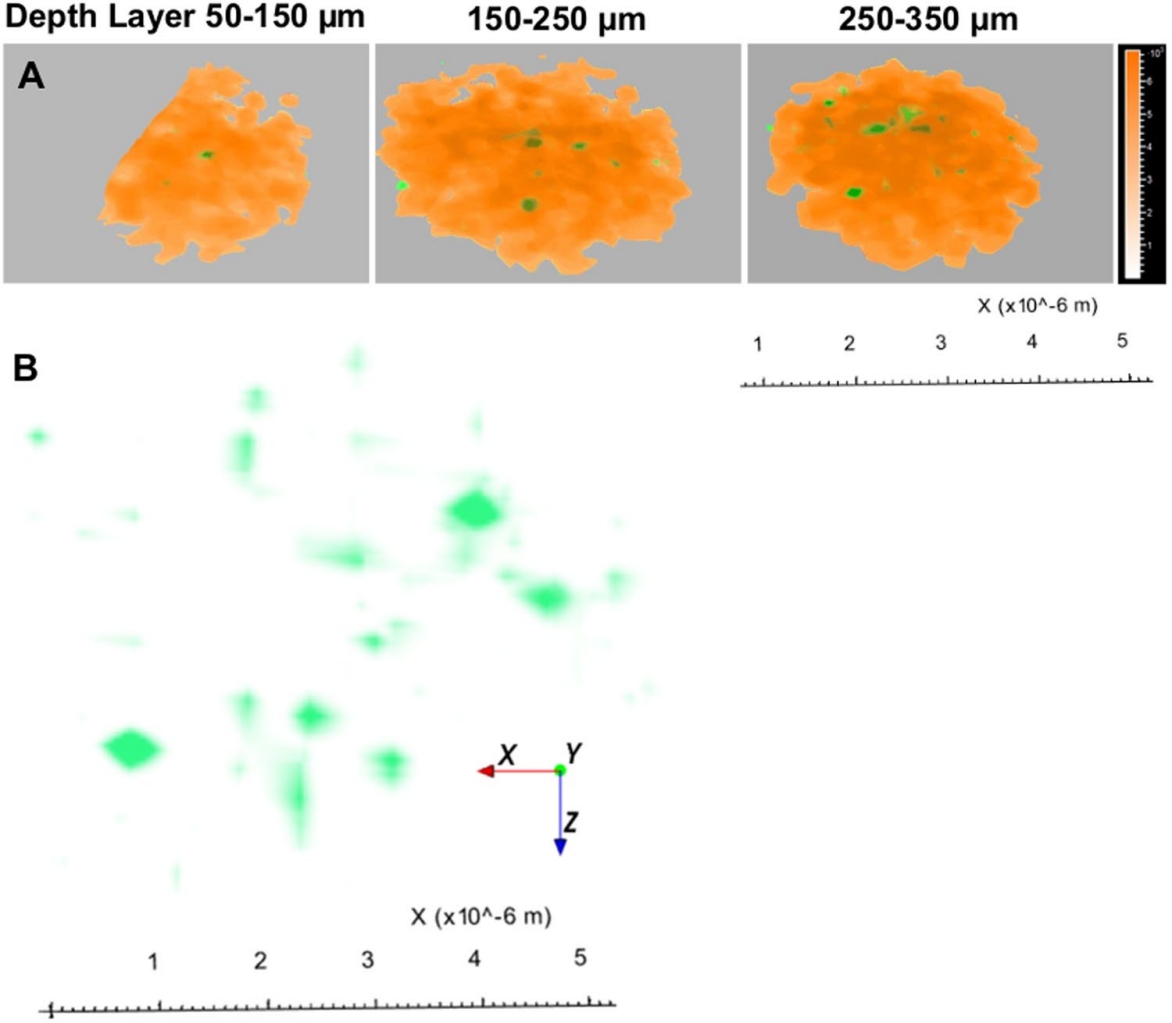
The spatial distribution of threonine-*O*-3-phosphate as agglomerates in a 3D reconstruction of a nanoparticulate cluster. The reconstruction shows the whole NP agglomerate and successive phosphate agglomerate distribution (depicted in green) in and around deeper layers of the spherical Au-NP. (**A**) Slicing the spherical NPs with different z-micro stacks and 3D reconstruction from top to bottom as shown in top view: Au particle reconstructed from Au^+^ signals (orange, solid); threonine-*O*-3-phosphate aureate (green). (**B**) Zoomed-in view spatially isolated threonine-*O*-3-phosphate aureate showing m/z 417.06 green signal within a single Au-NP cluster.

Figure 4 shows the 3D distribution of threonine-*O*-3-phosphate within a cluster of Au nanoparticles, indicating smaller sized threonine-*O*-3-phosphate (in green) agglomerates throughout the whole Au particle. Figure 4A shows a series of slices through a single nanoparticle cluster to a depth of 3 µm. Figure 4B only shows the 3D distribution of threonine-*O*-3-phosphate aureate (green) agglomerates from the Au particle. The data display a heterogeneous distribution of threonine-*O*-3-phosphate aureate agglomerates (green) within the biomineralized Au microenvironment.

### ToF-SIMS 3D imaging of human alveolar epithelial cells growing in a harsh ionic chemical environment

**Figure 5:**
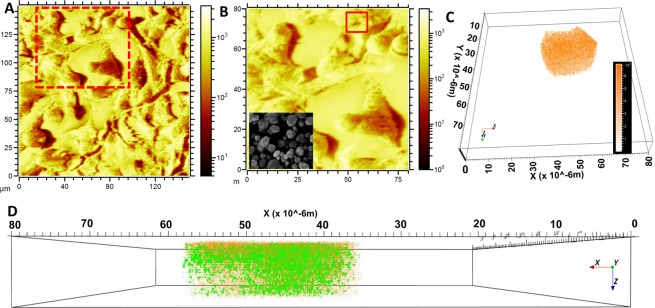
The constructed ion image reveals quasi spherical Au-NPs with increasing Au ions to 1 mM Au ions. (**A**,**B**) The topography is displayed by total ion reconstruction image of the A549 cell surface. Inset show SEM image of quasi-spherical nanostructure. (**C**) 3D reconstructed ion image of a single cluster with the Au^+^ signals (m/z 196.97) picked from biomineralized cell surface. (**D**) The zoomed in-depth 3D reconstruction overlay of cluster shown in (**C**) in side view of spatiotemporal Au^+^ signals (orange) with threonine-*O*-3-phosphate aureate (green).

**Figure 6:**
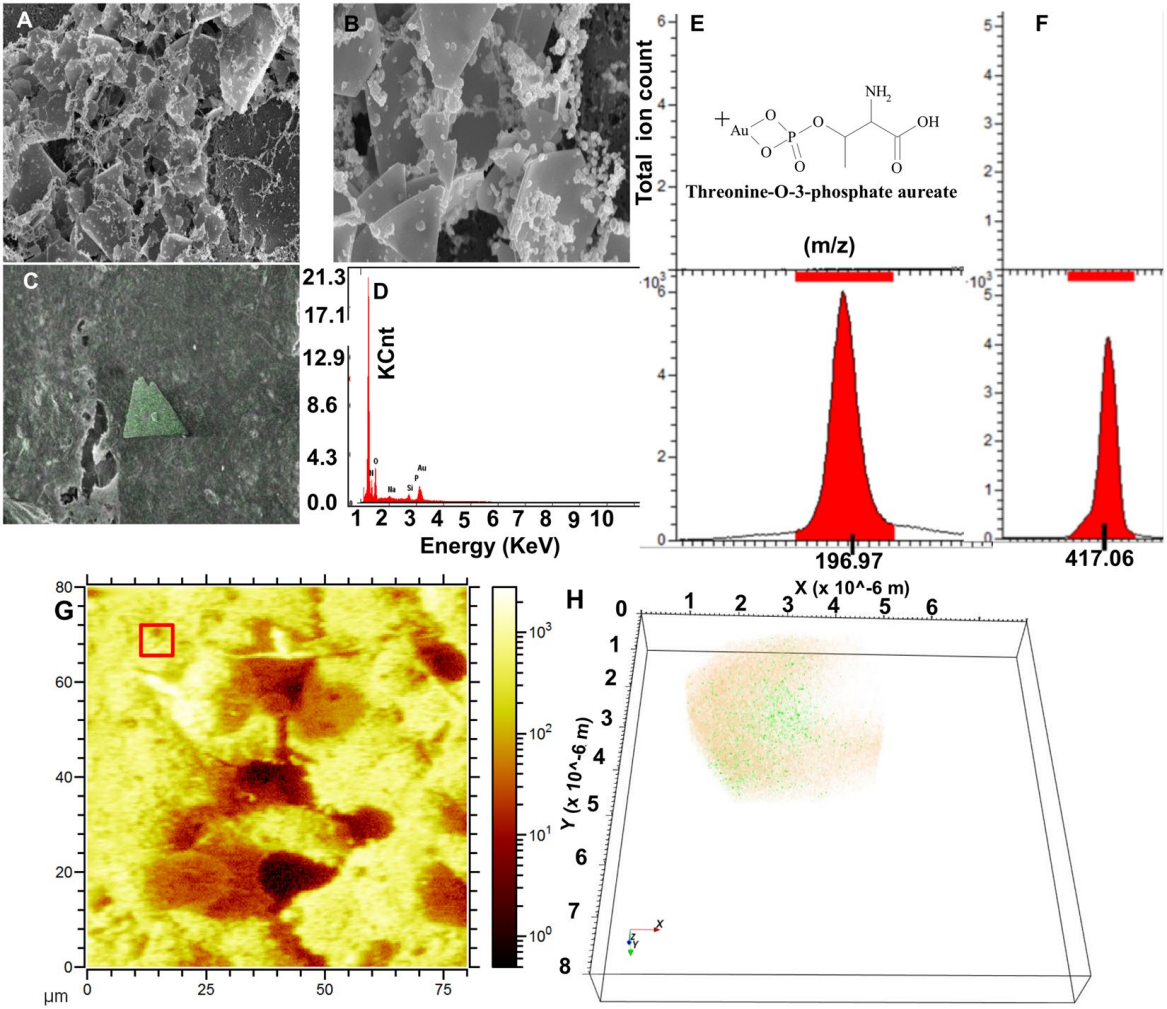
SEM and ToF-SIMS analysis of *de novo* biomineralized nanostructures at 2 mM Au ions: delta and rhombus shaped particles. (**A**–**D**) SEM and EDX chemical mapping to confirm anisotropic rhombus/delta shape Au nanoplate formation (Scale bar 1 micron). (**E**,**F**) The SIMS signals from Au^+^ (**E**) and threonine-*O*-3-phosphate aureate (**F**). (**G**) Reconstructed ToF-SIMS ion image from the A549 cell surface demonstrating spherical Au particles. (**H**) The 3D reconstruction and overlay of Au^+^ signal (orange) and threonine-*O*-3-phosphate aureate (green) from red square ROI from (**G**).

After doubling the Au ion concentration from 0.5 mM to 1.0 mM, no major change in the shape/size of the de novo synthesized particles was observed. The quasi-spherical NPs are visualized in the reconstructed ToF-SIMS ion images (see Fig. 5A,B). Figure 5C shows the ToF-SIMS ion image 3D reconstruction of a single *de novo* synthesized Au nanocluster from the Au^+^ signal. The 3D depth profiles in Fig. 5D reveal a similar chemical composition of the Au particle as seen in Fig. 4. Herein, the Au^+^ signal is shown in orange, whilst the threonine-*O*-3-phosphate signal is shown in green. Whilst in Fig. 4 threonine-*O*-3-phosphate agglomerates could be identified throughout the whole Au particle at low doses of 0.5 mM Au ions (Fig. 4D), threonine-*O*-3-phosphate was located only within the top 1.5 µm of the Au particle after incubation of cells with 1.0 mM Au ions (Fig. 5D). This biocomplexing homogeneity was more obvious when the Au^+^ concentration was increased to 2.0 mM. By means of SEM/EDX, we confirmed that rhombus/delta shape anisotropic structures are being formed (Fig. 6A–D). In addition, ToF-SIMS again revealed the presence of threonine-*O*-3-phosphate aureate and Au^+^ signals, respectively (Fig. 6E,F).

Extracting the threonine-*O*-3-phosphate aureate signals and surface rendering in 3D exhibits giant, buried biocomplexed Au interfaces precisely located within the 3D architecture, as shown in side view (Fig. 6G,H). The ionic Au structures are heavily interwoven with threonine-*O*-3-phosphate aureate (green) layers in a ROI of 5 × 5 microns. The role of threonine-*O*-3-phosphate aureate in the de novo synthesis of rhombus/delta shaped particles might arise due to close contacts between Au crystal plane and the peptide. The basis of anisotropy mainly involves the polar side chain of threonine, which homes a periodic structure of hydroxyl groups (–OH) into the regular lattice^6^.

### ToF-SIMS 3D imaging of human alveolar epithelial cell responses in harsh chemical environment with spherical NP seed as “solder” particles

**Figure 7:**
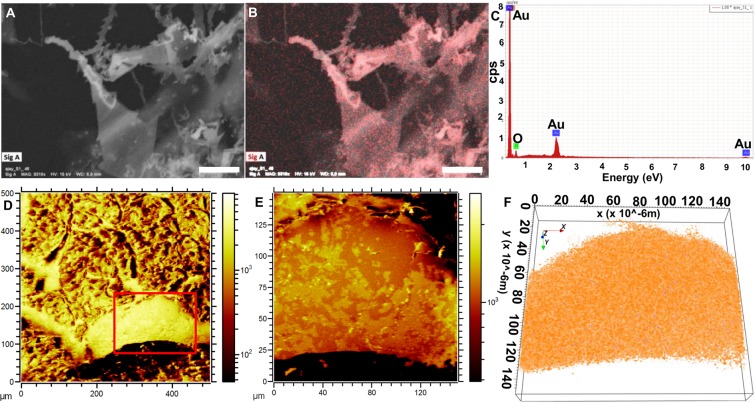
The *de novo* SEM and ToF-SIMS analysis of biomineralized long aspect ratio “nanoribbons” at 2 mM Au ions along with spherical Au-NP as seeds. (**A**–**C**) The SEM micrograph and EDX element mapping confirm the nanoribbon-like structure formation (scale bar 1 µm). (**D**–**F**) A qualitative view showing nanoribbon covered cell surface layers in ToF-SIMS topography (**D**,**E**) and overlapped ion signals in top view (**F**). The (**F**) is enlarged section from (**D**) shown with red square ROI. (**F**) demonstrates reconstruction of total ion count without background to show gold signals from (**E**).

We made further changes in the cellular microenvironment as described in schematic Fig. 1C. A549 cells were exposed to 2 mM Au ions supplemented with spherical NPs as seeds. In previous studies, it has been demonstrated that providing spherical NP seeds triggers long aspect ratio nanostructure synthesis in both biological and chemical synthesis routes^2,7^. First we confirmed the nanoribbon-like structure synthesis using SEM imaging and EDX analysis (Fig. 7A–C).

**Figure 8:**
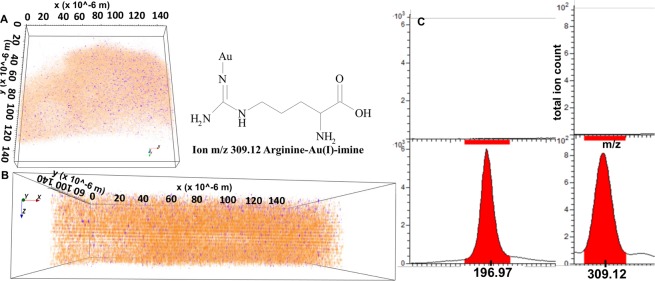
The ion image 3D reconstruction of Au^+^ signals (orange), and arginine-Au(I)-imine (blue) in top (**A**) view of the Au nanoribbon clusters on A549 cell. (**B**) The SIMS signals in side view from Au^+^ (orange) and arginine-Au(I)-imine (blue) showing that there is a change in chemical specificity of biocomplexation during Au mineralization supplemented with spherical NPs as ‘seed’ in cellular microenvironment. (**C**) The secondary ion signals from Au^+^ (m/z: 196.97) and arginine-Au(I)-imine (m/z: 417.06) (lower panel) and their comparison with untreated A549 cells (upper panel).

As shown with red pixels in Fig. 7B,C, Au signals clearly emerge from long aspect ratio nanoribbons embedded into the A549 cell. The presynthesized spherical NPs act as solder between triangular microplates, converting them into long nanoribbons^5^. Such nanoribbons act as a wave guide and facilitate faster propagation of Plasmon waves for photothermal therapy^5,8,9^. Starting from a large field of view of 500 × 500 µm, we focused on a smaller ROI of 120 × 120 µm where the cell membrane interacts with the nanoribbons as shown in Fig. 7D,F. The surface exhibits a wavy ribbon shaped contour as a result of the nanoribbons being distributed over the cell surface (compare 7.A with 7.D). Figure 8A,B further shows the ToF-SIMS ion reconstruction of an A549 cell culture exposed to 2 mM Au ions and 25 µL/mL Au nanoparticles (10 µg) as seeds. Figure 8A shows a3D cross sectional ion image of a *de novo* synthesized nanoribbon. Figure 8B shows side view of the Au signal in translucent orange and the chemical distribution of arginine-Au(I)-imine in yellow, indicating the presence of arginine-Au(I)-imine within the whole nanoribbon. Figure 8C shows the MS peaks associated with arginine-Au(I)-imine (m/z: 309.12) in A549 cells.

## Discussion

The knowledge about the exact chemical composition of biosynthesized nano- or microstructures could have a vital impact on understanding the synthesis yield enhancement of a single cell. The analysis of Au-NPs embedded in cancer cell surfaces is important for an exact assessment of the intracellular biokinetics and synthesis of NPs. It is a popular target for the SIMS-mediated detection of biomolecules secreted by mammalian cells^2^. In this report, for the first time to our knowledge, we demonstrate 3D molecular imaging of biomineralized Au particles of different shape using ToF-SIMS. The results demonstrate that mammalian A549 cells exposed to varying Au ion concentrations in serum-free media secrete specific Au binding/reducing biomolecules in response to the local microenvironment.

In A549 cell culture medium, we examined three different ionic environments (Fig. 1). Spontaneous deprotonation of HAuCl_4_ releases H^+^ ions into the cell environment at low Au ion levels. It causes reversible senescence and a pH shock to A549 cells as the mean pH shifts from neutral to acidic. As a strategy to optimize their survival, A549 cells then secrete buffer proteins, carbohydrates, and lipids^10^. The electron donors in metal binding amino acids^2,9^, carbohydrates, and stress proteins can reduce Au ions into nontoxic NP clusters (Fig. 3I–J). Interestingly when the extracellular microenvironment changes, the synthesized nanostructure shape shifts from spherical to irregularly shaped rhombic or triangular structures within the outermost 1.5 µm of the A549 cell’s membrane (Fig. 4). Interestingly, in highly ionic environments, when spherical particles are added into cell culture media to simulate seed mediated growth, the extracellular secretion of A549 cells changes from threonine-*O*-3-phosphate aureate (m/z: 417.06) to arginine-Au(I)-imine (m/z: 309.12), resulting in the biomineralization of Au ions into long nanoribbons (Figs. 7 and 8). The polar amino acid arginine promotes anisotropy in biomineralized Au nanostructures, as demonstrated in recent reports^9^. The production of these compounds may be a response of A549 cells to combat the Au ionic toxicity via transition-metal-catalyzed regiospecific amination as imine derivatives of L-arginine are better antioxidants in such circumstances^11,12^. The biological membrane associated surface proteins and dynamic topography of live A549 cells creates a confined diffusion boundary for nucleation and seed-mediated growth of long and versatile anisotropic nanostructures^13^.

In recent reports, molecular modeling and simulation studies indicate that Au binding polypeptides interact with Au^+^ nuclear lattices and exhibit specific sequence repeats, forming parallel and antiparallel β-sheet structures^8,14^. Threonine and serine polar side chains causes close contact between Au crystal planes and the peptide, which then rearranges the amino acid –OH groups into a regular lattice^6^. In anisotropic development, these Au binding peptides play a key role. Binding to other Au facets is not robust compared to crystal plane, primarily due to water-related molecular migration through the crystal atomic grooves, which decouples the polypeptide from the surfaces^14^. The Au nanoclusters adhere to the A549 cell surface via fibrils, which serve as a template for Au-NPs attachment. The application in this study of 3D molecular imaging has allowed for a better understanding of the biokinetics of *in-situ* reduction and further chemical analysis of the intracellular anisotropic micro/nanostructure distribution.

## Methods

### Materials

All reagents and chemicals were used as purchased without additional purification. Chloroauric acid (HAuCl_4_) was purchased from Sigma Aldrich (Cat # 254169-5 G) as an Au precursor. The cell counting Kit-8 (cat #CK04–20) was purchased from Tebu-bio, Offenbach, Germany. Cell culture materials (high glucose DMEM, fetal calf serum/FCS, penicillin/streptomycin, L-glutamine) were purchased from Gibco, Germany. All solutions were prepared in Milli-Q water (Millipore GmbH, Berlin, Germany) as the suitable solvent. Phosphate buffer saline (PBS) was purchased from Thermo Fisher Scientific (pH 7.4, Cat # 10010023). The UV/VIS absorption spectra were recorded with a Synergy UV-vis-near-infrared (BIOTEK, USA) spectrophotometer using Gen5 version 2.09 (https://www.biotek.com/products/software-robotics-software/gen5-microplate-reader-and-imager-software/technical-details/) analysis software.

### Cell culture/growth curves

Human alveolar basal epithelial cells A549 cells: A549 cells (ATCC Cat # CCL-18) were cultured in Dulbecco’s Modified Eagle Medium (DMEM) supplemented with 10% FCS, 1% penicillin/streptomycin (PAN) and 1% L-glutamine. Cells were passaged two times per week. Medium was changed every two or three days.

### Cell proliferation and cytotoxicity assay

The details of the cell proliferation and viability assays were adopted from our previous work^2,5^. A549 cells were seeded in 8 chambered microwells (Ibidi, Germany) with glass bottoms at a seeding density of 5 × 10^4^ cells mL^−1^ per well in complete DMEM (Life Technologies, Germany) and grown for 3 days at 37 °C in a humidified incubator containing 5% CO_2_ until they reached 70% confluency (~10^6^ cells per well). An overnight confluent culture of A549 cells was established in a 96 well plate. HAuCl_4_ in sterile PBS was directly added to the A549 confluent monolayer. After 48 hours of incubation, the cells were washed with sterile PBS (1×) to remove unreacted Au ions and free-floating nanoribbons. Biocompatibility was assayed with the cell counting Kit-8 (cat #CK04–20, Tebu-bio), which is a colorimetric assay for the determination of viable cells, cell proliferation, and cytotoxicity. The cell counting Kit-8 uses WST-8, a tetrazolium salt which produces the water-soluble WST-8 based colored formazan. Since this yellowish-to-orange colored formazan does not require further solubilization, no further solvent is required as compared to other assays such as MTT. Results are obtained after three steps: by (1) adding 10 μL of CCK-8 solution at 10:1 dilution in DMEM to each well of the 96 plate; (2) incubating the plate for 0.5–2 hours in the incubator; (3) using a microplate reader, measuring the absorbance at 450 nm. WST-8 is not cell-permeable and thus remains in the extracellular medium, which results in low cytotoxicity. We cross checked the cell viability with live cell permeable Calcein dye (Supplementary Figure S1).

### Synthesis of spherical nanoparticles via Au ion reduction

A549 cells were cultured to 100% confluency as packed monolayers. Subsequently, the culture media was replaced with PBS supplemented with sterile 0.5–1 mM HAuCl_4_ (final working dilution). This is term as condition (A-B) for nanoparticle synthesis as shown in Fig. 1. Cells were routinely assessed for nanoparticle synthesis and phenotypic changes with light microscopy^2^. Sample aliquots were taken for the confirmation of NP synthesis at different time points with UV/VIS spectroscopy, SEM/EDX analysis and ToF-SIMS imaging.

### Seedless/seed-mediated synthesis of anisotropic rhomboid microplates/long aspect ratio nanoribbons in A549 cell culture

As described, A549 cells were used to investigate nanoribbon synthesis in the presence of NP seeds under serum-free conditions. Filter sterilized 2 mM HAuCl_4_ solution in PBS (pore size 0.20 μm, pH 7.4) was used as a standard concentration for anisotropic NP synthesis. Under serum-free conditions, for seedless rhomboid particles, the growth medium of the confluent A549 cell monolayer was replaced with 2 mM HAuCl_4_ in sterile PBS (pH 7.4) from a 1 M stock solution. For seed-mediated synthesis, after 12 hours, biologically synthesized spherical Au-NPs (from synthesis scheme A) T25 culture flask with A549 and incubated at 37 °C and 5% CO_2_. The change in color from yellow to brownish on the cell surface was indicative of the formation of anisotropic nanoribbons after 24–36 hours. No color change was observed in the negative controls with live cells incubated in PBS without HAuCl_4_ or 4% paraformaldehyde in PBS fixed cells incubated with HAuCl_4_; or PBS incubated with HAuCl_4_ without cells. In PBS, Au solutions at different concentrations are shown in scheme A–D.

### Zeta potential and DLS measurement

The hydrodynamic size of the NPs was determined using a dynamic light scattering instrument (MÖBIUζ analyzer, Wyatt technology Germany) with an ATLAS pressurization system as described in our previous reports^2,5,9^. The details of method are described in our recent reports. The zeta potential was calculated from measurements of electrophoretic mobility performed by the same instrument.

### Scanning electron microscopy (SEM) and transmission electron microscopy (TEM) imaging

A Zeiss 55 Gemini Ultra SEM was used for imaging A549 cells with microplates/ nanoparticles/nanoribbons. An accelerating voltage of 3–5 keV and a high vacuum SE detector were used for imaging. The A549 cells were incubated with HAuCl_4_ different concentrations and conditions as explained in schematic Fig. 1. After the incubation period, cells were fixed in 2.5% glutaraldehyde in PBS for 30 min at 4 °C, rinsed with PBS, then gently washed with water. Cells were dehydrated in a series of graded aqueous ethanol (30, 50, 70, 90, and 100%, respectively) for 5 min each and 10 min in 100% ethanol^2,5,9^. Cells were further dehydrated and preserved using an automated critical point dryer (Leica EM, CPD 300). Wafers with cells were air dried followed by sputtering deposition of a 2–5 nm conductive nickel layer using a Leica coating system (Leica EM, ACE600).

### Sample preparation and ToF-SIMS analysis

After incubating A549 cells with different chemical environment of ionic Au precursor (AuCl_4_^−^ ions), the A549 cells were gently washed with PBS (pH 7.2) to avoid disturbing the intracellular and extracellularly synthesized micro/nanostructures on cell membranes^15–18^.Subsequently cells were fixed in 4% paraformaldehyde (PFA in PBS, Sigma Aldrich, Germany) for 30 min at room temperature. Post fixation, the cell layer surface was gently washed five times with reverse osmosis (RO) water to remove excess salt and cryofixation was done by plunge freezing immediately after washing. The plunge freezing cryofixation was achieved by manually immersing the sample in liquid propane cooled by quid nitrogen maintained at −196 °C. Propane enables a high cooling rate due to its high heat sink capacity, which helps to reduce the intracellular crystallization of water.

The distribution patterns of nanostructures within cell matrices (intra- and extracellular) or the identification of the biochemical composition was achieved by a ToF-SIMS 5 instrument (IONTOF GmbH Münster). The ToF-SIMS 5 was equipped with a liquid metal ion gun “30 keV Bi Nanoprobe” for deep profiling in combination with an argon gas cluster ion beam. The dual ion beam mode of the instrument enables multiple surface layer acquisition/analysis and 3D biochemical information reconstruction^19^. In this study, approximately 10 nm depth resolution and 80 nm lateral resolution per layer was achieved using the IONTOF instrument^20,21^. A 30 keV nano-bismuth primary ion beam source (Bi)x(y+) cluster with a Bi-Mn emitter as analyzing ion beam and a 20 kV argon cluster ion beam enabled to acquire ion images and 3D biomolecular depth profiling. Both sputter ion beam sources were mounted at 45 °C to the sample surface and an electron flood gun was integrated for charge compensation. The detailed protocols and 3D reconstruction of the NP distribution patterns from A549 cell systems are described by Sieg *et al*.^22^. We used absolute counts for the image analysis and results presentation throughout the paper^23–25^.

### Statistical analyses

All of the experiments shown in this study were independently repeated at least two times^2,5^. All the quantitative statistical analyses were performed by using Graphpad Prism 5 software version 5.02 (https://www.graphpad.com/scientific-software/prism/). Statistically significant differences were assigned with different alphabetic letters for p values less than 0.05. Two groups at the same time point were compared by a two-tailed Student’s t test. Multiple groups at the same time point were compared by one-way analysis of variance (ANOVA) with the Tukey-Kramer posthoc test^19–25^.

## Conclusions

This study reveals the capability of ToF-SIMS imaging to assess the chemical distribution of amino acid related chemical entities within de novo biosynthesized Au particles. It shows the possibility to visualize the chemical processes accounting for the *in situ* reduction of ionic Au into anisotropic structures. This is concept is demonstrated with A549 cells where a 3D reconstruction of a single Au particle can be made using ToF-SIMS 3D molecular imaging. Via ToF-SIMS imaging, this study shows that with increasing Au ion concentrations from 0.5 mM to 2.0 mM, there is an increased complexation between Au and biogenic compounds with the evolution of a homogenous porous architecture of threonine-*O*-3-phosphate aureate. The chemical specificity of threonine-*O*-3-phosphate aureate remains constant, but adding spherical NPs as seeds promotes synthesis of complexed Au in the form of arginine-Au(I)-imine. We here demonstrate a versatile aspect of ToF-SIMS molecular imaging. It represents a novel tool to probe the cellular biochemical microenvironment and could be extended further to elucidate the toxic effects of particles in the cell’s neighborhood.

## Supplementary information

Supplementary information.
