# Supplementary material for: ToF-SIMS 3D imaging unveils important insights on the cellular microenvironment during biomineralization of gold nanostructures
Source: Sci Rep. 2020 Jan 14;10:261. doi: 10.1038/s41598-019-57136-w (PMC6959255; doi:10.1038/s41598-019-57136-w)
Supplement: Supplementary file 1 — Supplementary information. [file 41598_2019_57136_MOESM1_ESM.pdf]

Supplementary Information

ToF-SIMS 3D imaging unveils important insights on the cellular microenvironment during biomineralization of gold nanostructures

Ajay Vikram Singh*, Harald Jungnickel, Lars Leibrock, Jutta Tentschert, Philipp Reichardt, Aaron Katz, Peter Laux, and Andreas Luch

Department of Chemical and Product Safety, German Federal Institute for Risk Assessment (BfR), Max-Dohrn-Strasse 8-10, 10589, Berlin, Germany

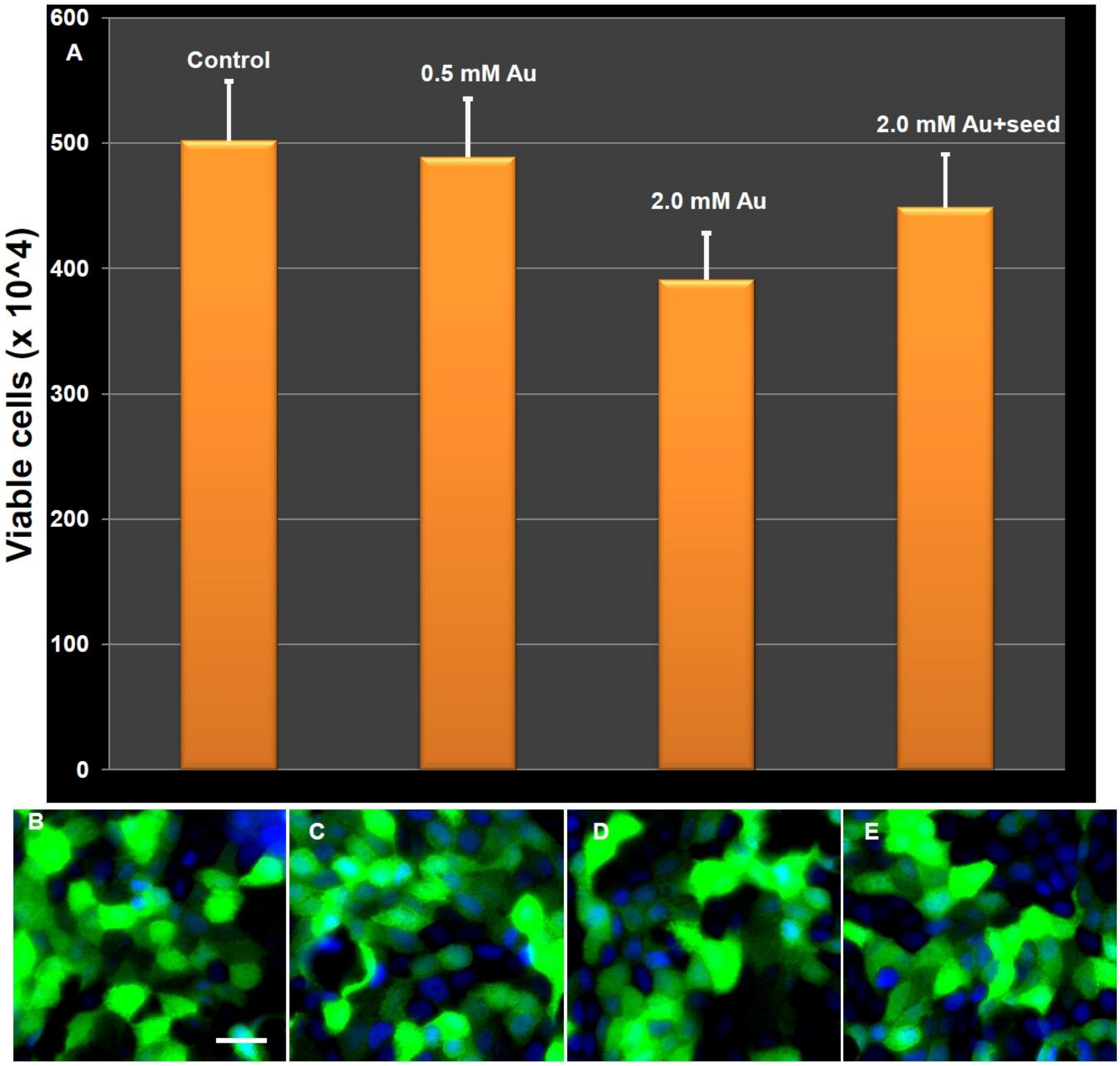

Figure S1. Cell viability of A549 culture with different ionic concentrations of gold. (A) Quantified viable cells. (B) Calcein (green) stained viable A549 with counter stained nuclei in blue (scale bar 25 µm).
